# The COVID-19 pandemic and the use of benzodiazepines and benzodiazepine-related drugs in Estonia: an interrupted time-series analysis

**DOI:** 10.1186/s13034-024-00757-5

**Published:** 2024-06-06

**Authors:** Katrin Kurvits, Karolin Toompere, Peeter Jaanson, Anneli Uusküla

**Affiliations:** 1 Institute of Family Medicine and Public Health, University of Tartu, 19 Ravila Street, 50411 Tartu, Estonia; 2 State Agency of Medicines, Tartu, Estonia; 3 Jaanson Psychiatric Center, Võru, Estonia

**Keywords:** Benzodiazepines, Z-drugs, COVID-19, Pandemic, Mental health, Interrupted time-series analysis

## Abstract

**Background:**

The COVID-19 pandemic has posed challenges that worsened people’s mental health. We explored the impact of the COVID-19 pandemic on the mental well-being of the population, as indicated by the prevalence rates of benzodiazepine and benzodiazepine-related drug (BDZ) use.

**Methods:**

This population‐based, time‐series analysis included all prescriptions of BDZs dispensed in Estonia between 2012 and 2021. The monthly prevalence rates of BDZ use were calculated. Autoregressive integrated moving average models with pulse and slope intervention functions tested for temporary and long-term changes in monthly prevalence rates after the onset of the COVID-19 pandemic.

**Results:**

Throughout the 10-year study period, a total of 5,528,911 BDZ prescriptions were dispensed to 397,436 individuals. A significant temporary increase in the overall prevalence rate of BDZ use in March 2020 (2.698 users per 1000, 95% CI 1.408–3.988) was observed, but there was no statistically significant long-term change. This temporary increase affected all the examined subgroups, except for new users, individuals aged 15–29 years, and prescribing specialists other than general practitioners and psychiatrists. The long-term increase in BDZ use was confined to females aged 15–29 years (0.056 users per 1000 per month, 95% CI 0.033–0.079), while no significant change was observed among males of the same age (0.009 users per 1000 per month, 95% CI – 0.017 to 0.035). Among females aged 15–29 years, a significant long-term increase in BDZ use was observed for anxiety disorders (0.017 users per 1000 per month, 95% CI 0.010–0.023), depressive disorders (0.021 users per 1000 per month, 95% CI 0.012–0.030), and other mental and behavioral disorders (0.020 users per 1000 per month, 95% CI 0.010–0.030), but not for sleep disorders (– 0.008 users per 1000 per month, 95% CI – 0.018–0.002).

**Conclusion:**

The COVID-19 pandemic led to a short-term increase in BDZ use immediately after the pandemic was declared. In the long term, young females experienced a sustained increase in BDZ use. The prolonged effect on girls and young women suggests their greater vulnerability. These results underscore the need to effectively address the long-term effects of the pandemic among youth.

## Introduction

The coronavirus disease 2019 (COVID-19) outbreak has introduced several stressors to mental health, including social isolation, stay-at-home orders, fear of contracting the disease, economic problems, and uncertainty about the future. Evidence from population surveys has shown elevated mental health concerns during the COVID-19 pandemic [1, 2]. In the UK, the prevalence of clinically significant mental distress increased from 18.9% in 2018–2019 to 27.3% in April 2020, one month after the lockdown [2]. Attempts to cope with these unexpected challenges may have led people to use anxiolytics, such as benzodiazepines, in response to the emergence of anxiety, fear, panic attacks, and sleep problems, whereas access to medical care was limited. Research on the effects of the COVID-19 pandemic, focusing on both the early stages and the later course, has documented mixed patterns of its impact on mental health [3].

Benzodiazepines and benzodiazepine-related drugs, so-called Z-drugs (BDZs), are among the most widely prescribed psychotropic medicines in developed countries, though they are commonly used in ways not supported by the evidence. BDZs are known to have high potential for treating dependence and addiction, and their use has been associated with a range of adverse effects, including cognitive and psychomotor impairment [4]. Therefore, their wider use is an important public health concern.

The existing evidence of the impact of the COVID-19 pandemic on the use of BDZ originates mainly from North America (USA [5–7], Canada [8]) or is constrained to specific population groups [9, 10], with conflicting results. A study conducted in the USA found an increase in the share of benzodiazepine dispensations to all controlled substances following the national emergency declaration [5]. However, in a Canadian study, no detectable deviations in benzodiazepine dispensing after the declaration of a national emergency were found [8]. A study assessing sex differences in the prescribing of BDZs among adults found an increase in Z-drugs in both men and women along with an increase in benzodiazepine prescriptions in women at the start of the COVID-19 pandemic [7]. Nevertheless, evidence is lacking in nationwide studies covering diverse population groups and all age groups, including children and adolescents. Examining the population-based trends of BDZs may provide essential information about health concerns and behaviors following significant events such as pandemics and their mitigation measures, as these medicines are often used to relieve acute symptoms of mental health.

This study aimed to explore the potential impact of the COVID-19 pandemic on BDZ use in a nationwide, population-based setting in Estonia.

## Methods

The study was conducted in accordance with the guidelines of the Declaration of Helsinki and the local data protection regulations. The study was approved by the Tartu University Research Ethics Committee. This study followed the Strengthening the Reporting of Observational Studies in Epidemiology (STROBE) reporting guidelines.

### Study design and data source

We conducted a retrospective, population-based interrupted time-series (ITS) analysis on the use of BDZs in Estonia between 2012 and 2021.

We obtained data from the Estonian Health Insurance Fund, which maintains a database of all outpatients (incl. persons without health insurance) prescriptions issued and dispensed in Estonia since 2011. For example, in 2021 10,740,326 prescriptions were dispensed to 903,329 inhabitants in Estonia [11].

This study included all prescriptions of BDZs (ATC groups N03AE, N05BA, N05CD, and N05CF) dispensed to Estonian residents from January 2012 to December 2021. For each prescription date of dispensing, data on patient and medication (incl. ATC code, amount dispensed), indication for use (ICD‐10 diagnosis code) and prescriber’s specialty were retrieved. Population data were obtained from Statistics Estonia [12].

In Estonia, BDZs are controlled prescription drugs belonging to Schedule IV of Narcotic Drugs and Psychotropic Substances List. Only physicians (regardless of profession) who have licensed to provide health care services can prescribe BDZs in Estonia. There is a quantitative limit on prescribing and dispensing BDZs: maximum of 60 tablets or 25 ml oral drops or 20 ampoules or 20 suppositories or 10 rectal tubes per prescription [13].

### Outcome measures and covariables

The monthly prevalence rates of BDZ use were calculated as the proportion of BDZ users per 1000 inhabitants.

Prevalence rates were further stratified by sex, age group (< 15, 15–29, 30–44, 45–59, 60–74, ≥ 75 years), indication of BDZ use, prescriber specialty (general practitioners (GPs), psychiatrists, or other specialties), user type category and BDZ group used (benzodiazepine or Z-drug alone or both simultaneously).

BDZ user type was categorized based on the previous year's usage: new (incident) user as no use or prior user as any use within the prior 365 days from every month analysed.

The indications for BDZ use were categorized into groups based on ICD-10 codes: sleep disorders (G47, F51), anxiety disorders (F41, F06.4, F40), depressive disorders (F32–F39, F06.32), other mental and behavioral disorders (all other F diagnoses), epilepsy (G40, G41) and other diagnoses.

### Statistical analysis

To evaluate the possible impact of the COVID-19 pandemic ITS analysis was conducted, fitting the ARIMA model on monthly prevalence rates (BDZ users/1000 inhabitants). The ARIMA model allows an examination of changes in prescribing while accounting for autocorrelation between consecutive monthly observations and seasonality [14].

The COVID-19 pandemic onset variable was set on March 2020, when the national emergency in Estonia was declared and the first lockdown was implemented. Thus, two periods were constructed: the prepandemic from January 2012 to February 2020 (98 data points) and the COVID-19 pandemic period from March 2020 to December 2021 (22 data points).

We tested temporary (pulse) and gradual (slope) intervention functions [15], as we hypothesized that restrictions of the COVID-19 pandemic may have had immediate temporary and gradual long-term effects rather than sudden, sustained change. To determine the parameters of differentiation and seasonality for the ARIMA models, an automated algorithm (auto.arima) was used, which repeatedly searches for the best model over a series of potential ARIMA models for the one with the lowest information criteria. The selected models were checked for white noise by using the residual plots and the Ljung-Box test.

Based on the final selected ARIMA models, the forecasts of predicted monthly prevalence rates in the absence of the intervention (the counterfactual) were generated to compare how the observed values differed from the forecast.

Analyses were conducted on the entire patient population of interest and for specific subgroups of interest. P values less than 0.05 were considered statistically significant. Analyses were performed using the statistical environment RStudio (R version 4.1.2) [16].

## Results

### Characteristics of BDZ use

In total, 5,528,911 BDZ prescriptions were dispensed to 397,436 individuals (260,591 females, 136,845 males) during the 10-year study period.

During the study period the mean monthly prevalence rate of BDZ use in Estonia was 28.0 users per 1000 inhabitants, being lowest in July 2012 and highest in March 2020, with 23.3 and 32.7 users per 1000 inhabitants, respectively. BDZ use is twice as common in females as in males, the mean monthly prevalence rates were 36.5 and 18.5 users per 1000, respectively. BDZ use increased with age, the mean monthly prevalence ranged from 0.3 users per 1000 among children under 15 years to 92.8 users per 1000 among those aged 75 years and older. The most frequent clinical indication for BDZ prescription was sleep disorders (with a mean monthly prevalence of 12.5 users per 1000), followed by anxiety, depressive, and other mental and behavioral disorders (with mean monthly prevalence of 3.5, 4.2, and 4.5 users per 1000, respectively), and epilepsy was rare (with a mean monthly prevalence of 0.3 users per 1000). BDZs are most frequently prescribed by GPs (with a mean monthly prevalence of 21.4 users per 1000), and more rarely prescribed by psychiatrists and other specialties (with mean monthly prevalence of 4.4 and 1.9 users per 1000, respectively).

### The possible impact of the COVID-19 pandemic

We observed a significant temporary (pulse) increase in the overall prevalence rate of BDZ use in March 2020 (2.698 users per 1000, 95% CI 1.408–3.988), but no significant change in the time trend (slope) from March 2020 until the end of the study period, December 2021 (p = 0.64) (Table 1).

**Table 1:** Interrupted time-series analysis results of the effect of the COVID-19 pandemic on the prevalence of benzodiazepine and Z-drug use in Estonia, 2012–2021

Benzodiazepine and Z-drug prevalence^a^	Change in pulse^b^ Estimate (95% CI)	Observed prevalence in March 2020	Predicted prevalence in March 2020	Percent change^d^ in March 2020 (%)	Change in slope^c^ Estimate (95% CI)	Observed prevalence in December 2021	Predicted prevalence in December 2021	Percent change^d^ in December 2021 (%)
Overall	**2.698 (1.408; 3.988)**	32.7	29.2	12.0	– 0.022 (– 0.116; 0.072)	31.4	31.6	– 0.3
Substance group								
Benzodiazepine only	**1.802 (1.159; 2.444)**	15.7	13.5	16.1	0.007 (– 0.050; 0.064)	14.3	13.8	3.6
Z-drug only	**0.634 (0.014; 1.254)**	13.2	12.5	5.2	– 0.025 (– 0.059; 0.008)	13.5	14.1	– 4.7
Benzodiazepine and Z-drug	**0.551 (0.377; 0.725)**	3.7	3.2	17.9	– 0.001 (– 0.014; 0.012)	3.7	3.6	4.2
User type								
Prior user	**2.545 (1.585; 3.505)**	28.8	25.4	13.2	– 0.020 (– 0.091; 0.052)	27.7	27.6	0.7
New user	0.138 (– 0.208; 0.484)	3.9	3.6	6.1	– 0.005 (– 0.042; 0.031)	3.7	3.8	– 0.2
Sex								
Female	**4.718 (2.888; 6.547)**	42.9	37.7	13.9	– 0.008 (– 0.129; 0.112)	40.7	40.3	1.0
Male	**1.222 (0.329; 2.116)**	21.2	19.9	6.8	– 0.016 (– 0.078; 0.046)	21.2	21.2	– 0.1
Age group								
< 15 years	**0.085 (0.014; 0.156)**	0.32	0.25	28.3	0.005 (0.000; 0.011)	0.36	0.25	45.1
15–29 years	0.255 (– 0.156; 0.665)	5.9	5.5	7.4	**0.032 (0.014; 0.050)**	6.8	5.9	15.8
30–44 years	**1.396 (0.504; 2.289**)	17.3	15.9	9.0	0.026 (– 0.032; 0.084)	17.3	16.3	5.8
45–59 years	**2.770 (1.328; 4.212)**	34.5	31.1	10.9	– 0.040 (– 0.163; 0.082)	33.0	33.5	– 1.6
60–74 years	**6.773 (4.212; 9.335)**	63.6	55.5	14.7	– 0.111 (– 0.342; 0.121)	60.8	61.4	– 1.0
≥ 75 years	**9.512 (4.378; 14.646)**	108.5	97.5	11.3	– 0.065(– 0.474; 0.343)	100.7	100.8	– 0.2
Indication								
Sleep disorders	**0.931 (0.284; 1.577)**	14.5	13.2	9.7	– 0.028 (– 0.075; 0.020)	14.6	15.1	– 3.2
Anxiety disorders	**0.620 (0.424; 0.816)**	4.6	3.9	18.0	–0.002 (– 0.014; 0.010)	4.2	4.2	1.3
Depressive disorders	**0.565 (0.347; 0.782)**	4.5	3.9	15.7	0.006 (– 0.007; 0.019)	4.1	3.9	5.5
Other mental and behavioral disorders	**0.414 (0.189; 0.639)**	5.2	4.6	14.0	–0.004 (– 0.017; 0.009)	4.7	4.6	1.4
Epilepsy	**0.030 (0.003; 0.057)**	0.29	0.26	9.8	0.000 (– 0.001; 0.001)	0.27	0.26	1.7
Other diagnoses	**0.226 (0.063; 0.389)**	3.6	3.3	8.7	0.000 (– 0.011; 0.011)	3.6	3.5	3.4
Prescriber’s specialty								
General practitioners	**2.547 (1.423; 3.671)**	25.6	22.9	11.6	– 0.002 (– 0.089; 0.086)	25.3	24.8	2.1
Psychiatrists	**0.579 (0.350; 0.808)**	5.2	4.5	13.2	– 0.027 (– 0.057; 0.003)	4.1	4.6	– 10.5
Other specialists	0.044 (– 0.121; 0.209)	1.6	1.5	1.4	**0.019 (0.011; 0.027)**	1.8	1.4	23.4

The bold font indicates statistical significance (p value < 0.05)

*CI* confidence interval

^a^Monthly prevalence—the number of users per 1000 inhabitants in a month

^b^Change in pulse—a temporary change in the prevalence in March 2020, when a national emergency due to the COVID-19 pandemic was declared

^c^Change in slope—a change per month in the prevalence from March 2020 to December 2021

^d^Percent change—a relative difference in observed compared to predicted prevalence

A significant temporary increase in the prevalence of BDZ use in March 2020 was observed across all subgroups examined, except for new users, users aged 15–29 years, and those prescribed BDZs by specialties other than GPs and psychiatrists (Table 1). For indications, a temporary increase in the use for anxiety and depressive disorders was the highest, at 0.620 users per 1000 (95% CI 0.424–0.816) and 0.565 users per 1000 (95% CI 0.347–0.782), respectively.

However, those aged 15–29 were the only age group that showed a significant increase in the slope (0.032 users per 1000 per month, 95% CI 0.014–0.050), which resulted in 15.8% more users by the end of the study period (observed in December 2021 6.818 users per 1000 vs. predicted in December 2021 5.888 users per 1000). Also, prescribing by other specialties increased in longer term (Table 1).

#### Adolescents and young adults aged 15–29 years

**Fig. 1:**
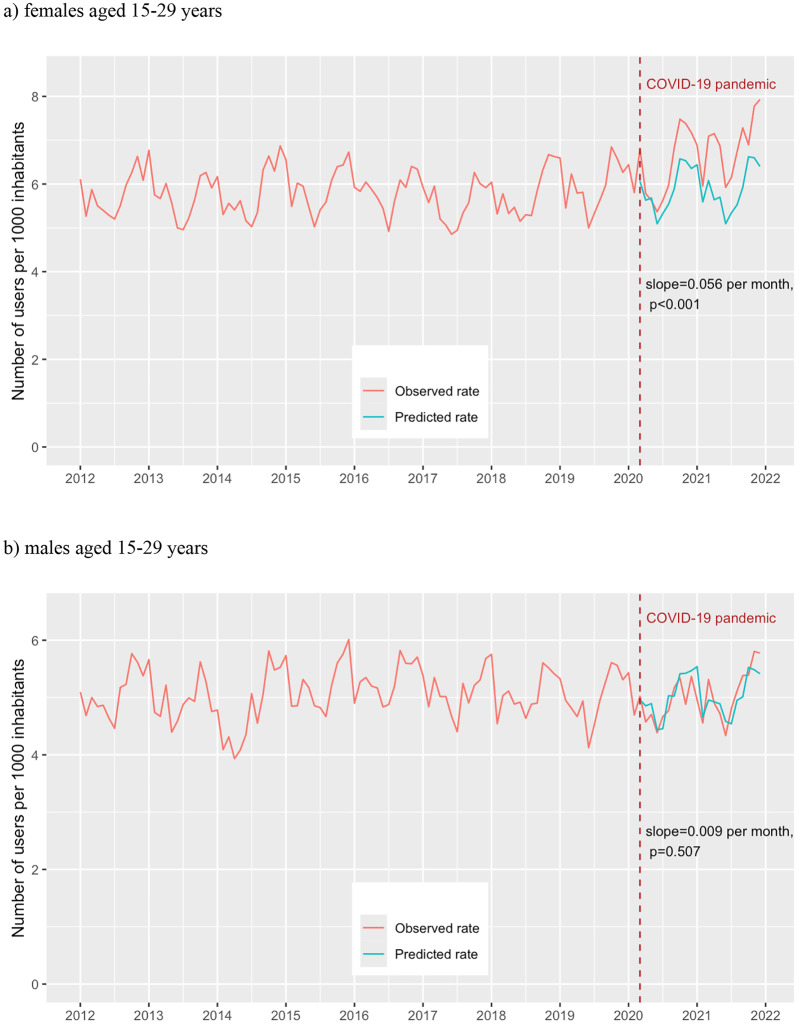
Interrupted time-series analysis of prevalence rate of benzodiazepine and Z-drug use among females (**a**) and males (**b**) aged 15–29 years in Estonia, January 2012 to December 2021 (red line—observed rate; green line—counterfactual predicted by ARIMA model in absence of the COVID-19 pandemic)

Adolescents and young adults aged 15–29 years were the only group that experienced significant gradual long-term effect in BDZ use. Stratifying by gender, the increase in slope was significant among young females (0.056 users per 1000 per month, 95% CI 0.033–0.079), but not among males (0.009 users per 1000 per month, 95% CI – 0.017–0.035) (Fig. 1). Additionally, a significant temporary increase in March 2020 was observed for females aged 15–29, but not for males of the same age.

Among females aged 15–29 with prior BDZ use, both a significant temporary increase (0.712 users per 1000, 95% CI 0.398–1.027) and a significant increase in the slope were observed (0.047 users per 1000 per month, 95% CI 0.027–0.067), which resulted in 28.0% more users by the end of the study period. However, among new users, only an increase in the slope was present (0.020 users per 1000 per month, 95% CI 0.012–0.028), which resulted in 20.7% more users by the end of the study period.

**Fig. 2:**
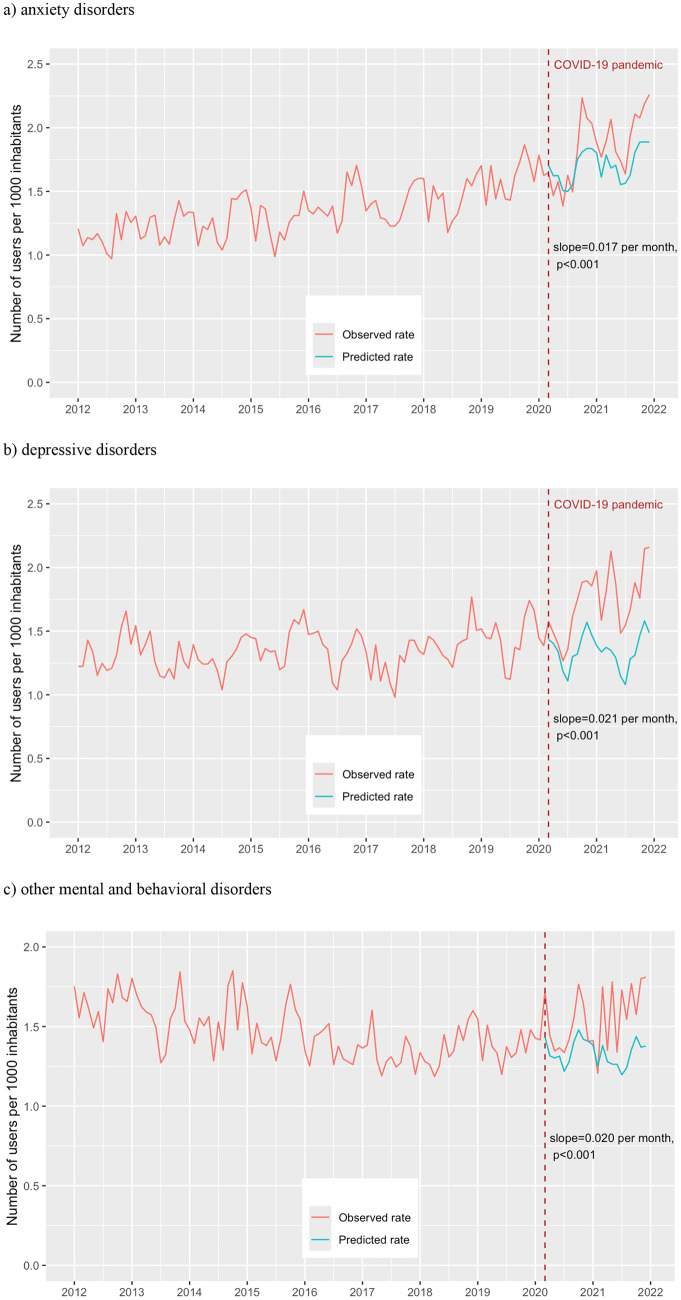
Interrupted time-series analysis of prevalence rate of benzodiazepine and Z-drug use among females aged 15–29 years in Estonia, January 2012 to December 2021, by main indications (red line—observed rate; green line—counterfactual predicted by ARIMA model in absence of the COVID-19 pandemic)

Among females aged 15–29, a significant increase in slope was observed for depressive disorders (0.021 users per 1000 per month, 95% CI 0.012–0.030), other mental and behavioral disorders (0.020 users per 1000 per month, 95% CI 0.010–0.030), and anxiety disorders (0.017 users per 1000 per month, 95% CI 0.010–0.023) (Fig. 2), which resulted in 45.1%, 31.5%, and 19.9% more users by the end of the study period, respectively. No significant change in use for sleep disorders was detected (– 0.008 users per 1000 per month, 95% CI– 0.018 to 0.002).

By prescribers’ specialties, a significant increase in the trend of prescribing BDZ for females aged 15–29 was observed only for psychiatrists (0.026 users per 1000 per month, 95% CI 0.016–0.037), but not for GPs or other specialties.

Among females aged 15–29, the increase per month in the prevalence of BDZ use after the COVID-pandemic onset was significant for the age groups 15–19 and 20–24, which resulted in 36.3% and 29.6% more users, respectively, by the end of the study period (Table 2).

**Table 2:** Interrupted time-series analysis results of the effect of the COVID-19 pandemic on the prevalence of benzodiazepine and Z-drug use among females aged 15–29 years in Estonia, 2012–2021

Benzodiazepine and Z-drug prevalence^a^	Change in pulse^b^ Estimate (95% CI)	Observed prevalence in March 2020	Predicted prevalence in March 2020	Percent change^d^ in March 2020 (%)	Change in slope^c^ Estimate (95% CI)	Observed prevalence in December 2021	Predicted prevalence in December 2021	Percent change^d^ in December 2021 (%)
Females 15–29 years	**0.562 (0.073; 1.052)**	6.8	6.1	12.2	**0.056 (0.033; 0.079)**	7.9	6.4	23.8
Prior user	**0.712 (0.400; 1.030)**	4.8	4.0	18.6	**0.047 (0.027; 0.067)**	5.5	4.3	28.0
New user	– 0.115 (– 0.500; 0.268)	2.0	2.1	– 2.6	**0.020 (0.012; 0.028)**	2.4	2.0	20.7
Sub age groups								
Females 15–19 years	– 0.237 (– 0.815; 0.341)	2.0	2.5	– 17.6	**0.051 (0.013; 0.088)**	4.0	2.9	36.3
Prior user	– 0.041 (– 0.425; 0.342)	1.1	1.3	– 14.5	**0.034 (0.009; 0.059)**	2.4	1.6	46.4
New user	– 0.218 (– 0.662; 0.227)	0.94	1.3	– 25.1	**0.016 (0.006; 0.027)**	1.6	0.9	75.0
Females 20–24 years	**1.018 (0.251; 1.784)**	7.6	6.4	20.1	**0.072 (0.015; 0.130)**	9.1	7.1	29.6
Prior user	**0.873 (0.322, 1.424)**	5.3	4.5	16.5	0.042 (– 0.025; 0.109)	6.0	4.8	24.8
New user	0.177 (– 0.487; 0.841)	2.4	1.9	26.2	**0.042(0.029; 0.055)**	3.2	2.0	57.7
Females 25–29 years	**0.977 (0.127; 1.826)**	9.9	8.9	11.5	0.035 (– 0.034; 0.104)	10.4	9.3	11.5
Prior user	**1.013 (0.339; 1.687)**	7.3	6.2	19	**0.051 (0.018; 0.085)**	7.9	6.2	27.3
New user	– 0.145 (– 0.660; 0.370)	2.6	2.7	– 4.7	0.005 (– 0.014; 0.024)	2.5	2.6	– 2.0

The bold font indicates statistical significance (p value < 0.05)

*CI* confidence interval

^a^Monthly prevalence—the number of users per 1000 inhabitants in a month.

^b^Change in pulse—a temporary change in the prevalence in March 2020, when a national emergency due to the COVID-19 pandemic was declared.

^c^Change in slope—a change per month in the prevalence from March 2020– December 2021.

^d^Percent change—a relative difference in observed compared to predicted prevalence.

A significant increase in the trend of new users was detected among females aged 15–19 and 20–24 years, which corresponded to a 75.0% and 57.7% increase in the prevalence of use at the end of the study period, respectively. However, among prior users, a significant increase per month was observed for females aged 15–19 years and 25–29 years, but not for those aged 20–24 years (Table 2).

## Discussion

This nationwide, population-based study examined the potential impact of the COVID-19 pandemic on the use of BDZ among all age groups in a Northern European country. Our study documents the impact of the COVID-19 pandemic from two different perspectives, identifying a greater impact among young people.

First, we observed an immediate increase in BDZ use after the COVID-19 pandemic outbreak and the declaration of restrictions to control it that affected people of all ages, genders, and experience with BDZ use. A similar temporary increase was observed by Jones et al. [6], who reported an increase in the monthly number of patients dispensed antidepressants and benzodiazepines in March 2020, which returned to forecasted levels in April–May 2020. Assessing disease prevalence is crucial for understanding the health needs of populations, and medication data often used as a proxy for chronic disease prevalence [17]. The temporary increase in BDZ use observed in our study can be considered a surrogate indicator of newly emerged or deteriorated mental health problems in the population. Similarly to what has been described in other countries, the risk of depression and anxiety increased considerably in Estonia during the pandemic years compared to 2019 [18]. Our findings highlight the vulnerability of individuals with pre-existing mental health disorders and/or chronic illnesses. We observed a significant temporary increase among prior users, but not in overall incident use. This temporary surge may be attributed partially to individuals already using these medicines, who began stockpiling when the restrictions were imposed. Additionally, the temporary increase may be linked to economic hardships arising from the pandemic outbreak. An Estonian study revealed that unemployment was associated with a heightened risk of depression within the first months of becoming unemployed, as well as an increased risk of anxiety both immediately and one year later [18].

Second, we identified a prolonged effect on the young female population that persisted throughout the pandemic years. No such effect was found among older age groups. This effect among young people was driven by both an increase in BDZ use among experienced users and an influx of new users. However, such a change was not present among males of that age. Similarly, Milani et al. [7] also showed that compared with men, women had a higher rate of prescriptions for benzodiazepines, Z-drugs, and SSRIs/SNRIs and had greater changes in prescription rates over time, suggesting that the gender disparity in mental health has been exacerbated by the pandemic. Jacques-Avinõ et al. [19], reported a higher proportion of anxiety and depression in younger people (18–35 years), especially in women. Poorer mental health was mainly related to fear of COVID-19 infection with higher anxiety levels, and worsened economy with higher levels of depression in women [19]. Also in our study, we observed a significant persistent increase in use for depression and anxiety disorders, but not for sleep problems. Sleeping disorders are the single most frequent reason for BDZ prescriptions in Estonia, however prescribing for this did not increase during the COVID-19 pandemic.

In our study, girls aged 15–19 years seemed to be most affected, as a significant increase in the longer term was detected both for prior and incident users. Similarly, Bliddal and colleagues [20] reported that Danish youths experiencing increased rates of incident psychotropic medication use and psychiatric disorder diagnoses during the COVID-19 pandemic, which was most pronounced among those aged 12–17 years. Although mental health among young European people had already deteriorated before the pandemic, numerous studies have observed sharp increases in the rates of depression, tension, and anxiety among children and adolescents during the COVID-19 crisis. A meta-analysis [21] of 29 studies worldwide covering approximately 80,000 children and adolescents under 18, suggested that the rates of clinically significant depression and generalized anxiety symptoms doubled during the pandemic, with one in four youth experiencing depression and one in five experiencing anxiety. Higher rates of anxiety and depression were noted among girls and young women [21]. Also, in Estonia, 15–17-year-old girls are reported to have a greater risk of mental health disorders than boys of the same age or girls of older age [18]. Children are primarily susceptible to cognitive and communicative vulnerability. The mental health distress of young people during the COVID-19 pandemic can be explained by the learning disruption caused by school closures and coping with remote learning, decreased social interactions due to physical social distancing, the closures also meant disruption of leisure activities, including physical activity. A systematic review and meta-analysis [22] unveiled notably heightened general anxiety symptoms during periods of school closure and other restrictive measures among children and adolescents. The school closures during the first waves of the COVID-19 pandemic significantly disrupted the lives of adolescents in Estonia. The number of days when lower secondary and upper secondary general schools were fully closed between January 2020 and May 2021 far exceeded the OECD average in Estonia [23].

The finding that older adults experience less psychological distress than younger adults has long been termed ‘the well-being paradox’ because older adults report better emotional well-being despite being more likely to experience health problems, physical limitations, and loss of loved ones [24]. Also during the pandemic, access to medical care was limited, which impeded immediate professional help when problems arose. Estonia has been estimated to have the greatest unmet need for health care in the European Union [25]. Even before the pandemic, the queues for mental health services were unacceptably long in Estonia, due to the acute shortage of psychiatrists, as well as clinical psychologists, school psychologists and mental health nurses [26]. The GPs’ role as the doctor of first contact care for psychological problems has increased in Estonia [27], but the workforce shortage is evident also for primary care in Estonia. Furthermore, the workload in primary care increased greatly during the pandemic. Estonian GPs have expressed that their readiness to manage patients with psychological problems, yet many of them require additional training [28].

Due to the risk of developing dependence and severe adverse effects, the use of BDZ should be closely monitored to avoid causing more problems and harm [18]. Considering the increased use of BDZs during the pandemic, it is necessary to apply adequate complementary nonpharmacological measures. Youth mental health requires continued attention and support even after the pandemic has ended. The gap between the psychotherapy resources available and the resources urgently needed can foster the use of treatments, such as BDZs, that provide immediate effects but are not in the patient's best interest in the long term. Failure to address psychological problems properly and timely at a young age may have negative consequences later in life.

We acknowledge that our study has some limitations. First, our study is susceptible to misclassification bias, which may have resulted in an overestimation of BDZ use. It is possible that not all medicines purchased were consumed, and some may have been shared with relatives or sold. Second, our analysis relied on routinely collected data, which may have limited the availability of certain variables for analysis. Additionally, the generalizability of our results is restricted to regions with similar health systems. Nevertheless, a major strength of our study is the large, non-selected, nationwide population sample. Furthermore, our investigation allowed for a detailed exploration of the relationship between BDZ use and mental health disorders. Unlike several other prescription registries lacking data on indications for use [29], these data were available to us.

The primary underlying assumption when utilizing prescription data to represent a disease is that individuals who have a particular illness will be prescribed specific medications from a designated list. We recognize the intricate interplay between patient expectations regarding medication and how doctors perceive these expectations [30].Nonetheless, there is substantial evidence supporting the reliability of prescription data in terms of tracking disease prevalence and incidence [31–34], responsiveness to shifts in prescription trends, and reduced susceptibility to over-reporting compared to methods such as self-reporting for conditions that are clinically diagnosed or have vague definitions, such as migraine, depression, or anxiety [35].

## Conclusion

This nationwide, population-based study revealed a significant temporary increase in BDZ use in Estonia following the emergence of the COVID-19 pandemic. However, a more concerning finding was the persistent increase in the use of BDZ among young females, highlighting the disproportionate impact of the pandemic on their mental health. These results underscore the need to effectively address the long-term effects of the pandemic on benzodiazepine and Z-drug use, given the potential for addiction and associated public health implications. Additionally, these findings provide background information for complex approaches and to support better decision-making in similar situations in the future.

## Data Availability

The data underlying the results presented in this study are subject to restrictions and are not publicly available but can be obtained by request from the Estonian Health Insurance Fund (https://www.haigekassa.ee/andmeparingud).
